# Effect and safety of Chinese patent medicine capsules for recurrent angina pectoris after percutaneous coronary intervention: A protocol for systematic review and meta-analysis

**DOI:** 10.1097/MD.0000000000023287

**Published:** 2020-12-04

**Authors:** Yize Sun, Zheyi Wang, Chao Wang, Zhuoran Tang, Jinyu Shi, Haibin Zhao

**Affiliations:** a Beijing University of Chinese Medicine; b Oriental Hospital, Beijing University of Chinese Medicine, Beijing, China.

**Keywords:** angina pectoris, Chinese patent medicine capsule, meta-analysis, percutaneous coronary intervention, protocol, systematic review

## Abstract

**Introduction:**

Recurrent angina pectoris after percutaneous coronary intervention (PCI) is a common clinical syndrome, which seriously reduces the quality of life and health of patients, increases medical costs, and causes the risk of cardiogenic death. The efficacy of various western medicine improving angina symptoms has not been fully confirmed at the moment, whereas Chinese patent medicine capsules (CPMC) have been generally used in clinical practice due to the therapeutic efficacy and safety. This study evaluates the efficacy and safety of CPMC for stable angina after PCI, designed to provide more evidence for clinical treatment.

**Methods:**

This protocol was based on the previous reporting items. We will search 3 English databases (PubMed, Excerpta Medica Database, and the Cochrane Library) and 3 Chinese databases (China Network Knowledge Infrastructure, Wan Fang Database, and Chinese Biomedicine) until January 2020. RCTs to evaluate the efficacy and safety of CPMC for recurrent stable angina pectoris after PCI will be included. The primary outcome will be assessed by major adverse cardiovascular events and angina attack frequency. We will use the criteria provided by Cochrane risk of bias tool for quality evaluation and risk assessment, and use the Revman 5.3 for meta-analysis.

**Ethics and Dissemination:**

Ethical approval is not required for systematic review and meta-analysis. The results of this review will be disseminated in a peer-review journal.

**PROSPERO registration number:**

CRD42020164005.

## 1 Introduction

Chronic diseases are causing an increasing number of deaths worldwide, and ischemic heart disease remains the leading killer in the past decade, according to a report on the world health organization's website.^[1]^ Percutaneous coronary intervention (PCI) can reduce cardiovascular mortality and the risk of recurrence of myocardial infarction.^[2]^ Nevertheless, it is still faced with many challenges, of which recurrent angina is particularly difficult to manage. The TRANSLATE-ACS Study, a longitudinal study of myocardial infarction (MI) patients treated with PCI at 233 US hospitals from 2010 to 2012, observed that nearly 30% of patients undergoing PCI for MI reported angina 6 weeks later, and one-third of these patients continued to have angina at 12-month follow-up.^[3]^ Angina post-PCI even approximately involved from one-fifth to one-third of patients undergoing repeated revascularization at 1-year follow-up.^[4]^ The occurrence of ≥1 episode of angina on a weekly basis is associated with worse quality of life and greater physical limitations.^[5]^ Additionally, health care costs in patients with persistent or recurrent angina may be twice as people who had no symptoms after PCI.^[6]^

The pathophysiology of angina after PCI is complex, comprising both structural and functional alterations. Structural causes include in-stent restenosis (ISR), stent thrombosis, progression of atherosclerotic disease in other coronary segments, incomplete revascularization, presence of myocardial bridges, and diffuse atherosclerosis without focal stenosis, while functional causes mainly include vasomotor abnormalities of epicardial coronary arteries and coronary microvascular dysfunction.^[7,8]^ Although recurrent angina post PCI differences from other angina in terms of pathophysiological mechanism, current guideline or consensus does not distinguish it from other treatments.^[9]^ Some literatures reported have discussed the progress of treatment. In case of structural causes, the need for further revascularization should be carefully evaluated. As for functional causes, the therapeutic approach targets the underlying pathophysiological mechanism, covering traditional and nontraditional antiischemic drugs, the former recommending Beta-blockers, nitrates, angiotensin-converting enzyme inhibitor, and Ca-antagonists, the latter including ranolazine, xanthines, nicorandil.^[4,10,11]^ Despite some evidence supporting specific interventions for this type of angina pectoris, this appears not to be sufficient because of the absence of large prospective trials. Exploring novel and effective strategies for this population is urgently needed.

Traditional Chinese medicine (TCM) can not only directly reduce the incidence of angina and improve the quality of life of patients after PCI,^[12,13]^ but also has a beneficial effect on reducing the ISR rate and the degree of restenosis.^[14]^ Chinese patent medicine capsules (CPMC), as the main dosage form of oral preparation, are favored by the majority of patients because of diverse advantages, such as easy to carry, convenient to take, odor isolation, high bioavailability, and time release,^[15]^ widely used in the treatment of recurrent angina pectoris. In recent years, high-quality randomized controlled trials (RCTs) reporting the application of CPMC are increasingly being completed, which seem to have potential efficacy, yet there is incomplete evidence currently to assess the efficacy.^[16,17]^ Therefore, we made a systematic review and meta-analysis of published RCTs to scientifically and objectively evaluate the effect of Chinese patent medicine capsules. The review aims to shed new light on the treatment methods for angina post PCI with reduced side effects.

## 2 Methods

### 2.1 Study registration

This systematic review protocol has been registered in PROSPERO (registration number: CRD42020164005), and has been checked with Preferred Reporting Items for Systematic review and Meta-Analysis Protocols (PRISMA-P) checklist.^[18]^

### 2.2 Inclusion criteria

#### 2.2.1 Type of studies

Only RCTs to evaluate the efficacy and safety of CPMC for recurrent angina pectoris after PCI will be included. The language is limited to Chinese and English.

#### 2.2.2 Type of participants

Participants below 80 years old will be recruited who are clinically diagnosed with stable angina after PCI, according to “2013 ESC guidelines on the management of stable coronary artery disease.”^[19]^ It should be noted that only those graded on a scale of I to III in classification of angina severity according to the Canadian Cardiovascular Society (CCS) will be included in this study. CCS is widely used as a grading system for angina to quantify the threshold at which symptoms occur in relation to physical activities.^[20]^ No restriction on race and irrespective of gender.

#### 2.2.3 Type of intervention

Treatment group employs CPMC, in combination with control group treatments, while control group uses routine therapies alone, or with placebo capsules. Routine therapies cover antiplatelet agents, beta-blockers, calcium-channel blockers, lipid-lowering medication, and other oral medicine recommended by guidelines.

#### 2.2.4 Outcomes

##### 2.2.4.1 Primary outcomes

The primary outcomes will be evaluated by angina attack frequency and major adverse cardiovascular events (MACE). In this study, MACEs were defined as cardiac death, MI, target lesion revascularization, and new onset of congestive heart failure. All the outcomes were independently adjudicated by 2 cardiologists.

##### 2.2.4.2 Secondary outcomes

The secondary outcomes will be assessed by adverse events and Seattle angina questionnaire (SAQ), which has been extensively used in clinical trials and registries to quantify patients’ symptoms, functional status, and quality of life.^[21]^

### 2.3 Search strategy

#### 2.3.1 Electronic searches

We will search the following 6 databases until January 2020: PubMed, Excerpta Medica Database, the Cochrane Library, China National Knowledge Infrastructure, Wan Fang database, and Chinese Biomedical.

#### 2.3.2 Searching other resources

Meanwhile, we will search Chinese Clinical Trial Registry and the US National Institutes of Health Ongoing Trials Register for any related ongoing or unpublished trials.

#### 2.3.3 Search strategy

Mesh term will be applied into databases. The search strategies are presented as follows:

#1 Search (percutaneous coronary intervention [MeSH Terms]) OR (PCI [Title/Abstract]) OR (revascularization [Title/Abstract]) OR (percutaneous coronary revascularizations [Title/Abstract]) OR (coronary revascularization [Title/Abstract])
#2 Search (angina pectoris [MeSH Terms]) OR (stable angina pectoris [Title/Abstract]) OR (chronic stable anginas [Title/Abstract])
#3 Search (capsule [Title/Abstract]) OR (collocystis [Title/Abstract]) OR (encapsulant [Title/Abstract])
#4 Search (traditional Chinese medicine [MeSH Terms]) OR (TCM [Title/Abstract]) OR (Chinese medicine [Title/Abstract]) OR (Chinese herbs [Title/Abstract])
#5 Search (“randomized, controlled trial” [MeSH Terms]) OR (“randomized controlled trial” [Title/Abstract]) OR (“clinical study” [Title/Abstract]) OR (“clinical trial” [Title/Abstract])
#1 AND #2 AND #3 AND #4 AND #5

### 2.4 Data collection and analysis

#### 2.4.1 Study selection

**Figure 1:**
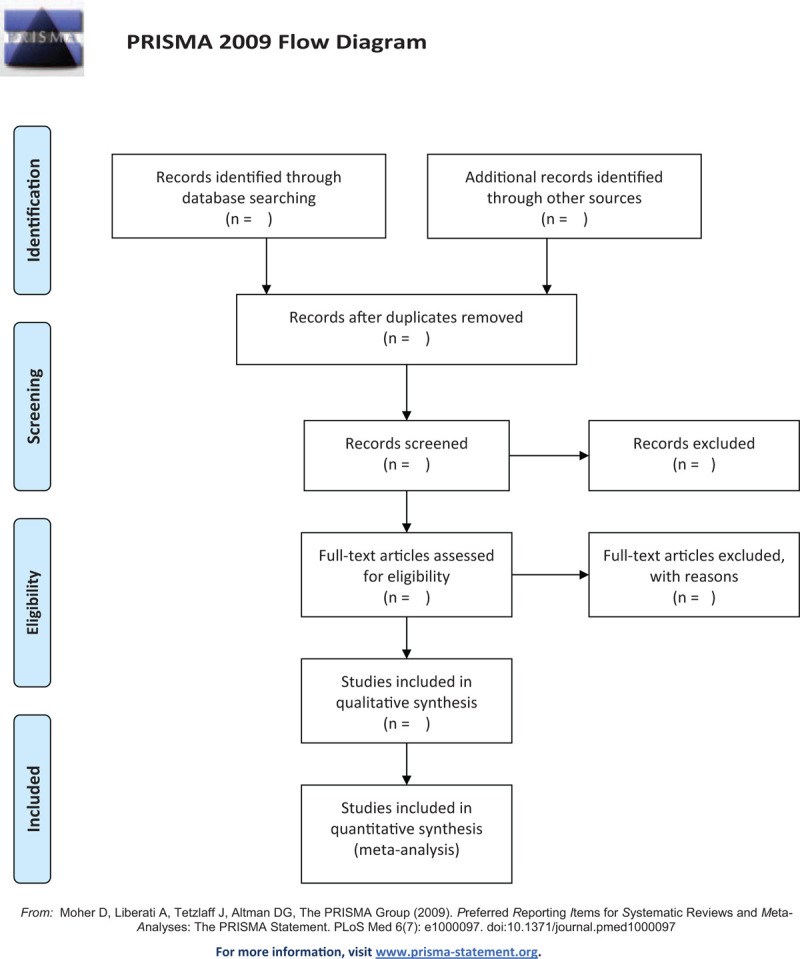
PRISMA flow diagram.

The articles from multiple databases will be imported into endnoteX9 software to delete the duplicate studies. Two investigators will independently scan the title and the abstract of every record to exclude articles inconsistent with the study. The full text of the qualified literature will be investigated to further assess and determine whether the trials meet the inclusion criteria. A third author will be consulted for an expert opinion in the event of any contradiction. Details of study selection process is shown in a PRISMA flowchart (Fig. 1).

#### 2.4.2 Data extraction

Based on the standardized data extraction form from Cochrane Handbook, 2 investigators will separately extract the following detailed information:

1. general information: title, authors’ name, journal, publication year, country.
2. participants: gender, age, duration of disease, sample size.
3. interventions: capsules composition, dose, frequency of administration.
4. Outcomes: angina attack frequency, MACE, adverse events, SAQ.

#### 2.4.3 Risk of bias assessment

Two authors will independently assess the methodological quality of studies included by using the Cochrane risk of bias tool for randomized trials.^[22]^ The following 7 domains will be concluded: generation of the random sequence, allocation concealment, blinding of participants and investigator, blinding of outcome assessors, incomplete outcome data, selective reporting, and other biases. The methodological qualities of the included studies will be classified as being “low” (representing a low risk of bias), “high” (for a high risk of bias), or “unclear” (for a medium or unknown risk of bias).

#### 2.4.4 Data synthesis

We will adopt RevMan 5.3 software to perform the analysis of the data. In terms of dichotomous variables, we will select relative risk with 95% confidence intervals (CI). As for the continuous variables, we select weighted mean difference with 95% CI. *P* < .05 is considered statistically significant.

#### 2.4.5 Assessment of heterogeneity

We will calculate *χ*^2^ test and I^2^ statistic to assess the existence and degree of heterogeneity. Under the circumstance of I^2^ < 50%, *P* > .01, fixed-effects model will be applied, considered as no heterogeneity. Otherwise, the fixed effects model will be employed. If there is significant heterogeneity between studies, we will explore the root causes of heterogeneity from multiple aspects, such as drug dose and follow-up time. If necessary, sensitivity analysis or subgroup analysis would be used to elucidate the heterogeneity.

#### 2.4.6 Subgroup analysis

Based on the characteristics of the study, we will perform a subgroup analysis to probe underlying sources of heterogeneity, including sample size, treatment duration, TCM treating principle, and other relevant parameters.

#### 2.4.7 Sensitivity analysis

We will conduct sensitivity analysis to assess the robustness and reliability of the results. By means of changing inclusion criteria, excluding low-quality studies or studies with small sample sizes, changes in merge indicators will be observed.

#### 2.4.8 Publication bias

When studies included in meta-analysis are more than 10, the funnel plot would be generated to evaluate whether there is publication bias. Funnel map asymmetry indicates publication bias. If needed, we will put Begger tests in STATA 12.0 software into use.

#### 2.4.9 Evidence assessment

According to grading of recommendations assessment methods, we evaluate the strength of evidence for each outcome rating as “high,” “moderate,” “low,” “very low.”

## 3 Discussion

Angina pectoris is a common complication after PCI, involving several mechanisms, among which microvascular angina was identified as important functional mechanism and requires drug intervention clearly.^[7,23,24]^ Despite several drugs for coronary microcirculation disorders, Western medicine treatment is still mainly focused on routine secondary prevention of coronary heart disease. The theory of collaterals, anatomically similar to blood vessels, as an important theory of TCM, plays an important role in guiding syndrome differentiation, diagnosis, and medication, especially in the cardiovascular and cerebrovascular field.^[25,26]^ Based on the collateral theory, many Chinese medicine are compatible reasonably to be prescribed and applied broadly and efficaciously in clinical practice. CPMC have good effect in interfering with functional mechanism and treating angina pectoris, widely used in China, which have become an important topic in medical research, deserving attention and promotion, but its efficacy and safety have not been systematically evaluated. Therefore, we will use systematic review and meta-analysis to evaluate the efficacy and safety of CPMC, expecting that the review could provide more options for the treatment of angina patients post PCI.

## Author contributions

HZ conceived of the study; ZT and JS searched the literature; ZW and YS performed the data analysis; CW conducted methodological supervision; YS drafted the manuscript; All authors have approved the final manuscript.

**Conceptualization:** Yize Sun.

**Data curation:** Zhuoran Tang.

**Formal analysis:** Yize Sun.

**Methodology:** Zheyi Wang, Jinyu Shi.

**Resources:** Chao Wang.

**Software:** Zheyi Wang.

**Supervision:** Haibin Zhao.

**Writing – original draft:** Yize Sun.

**Writing – review & editing:** Yize Sun.
